# Up-regulation of long non-coding RNA *SPRY4-IT1* promotes tumor cell migration and invasion in lung adenocarcinoma

**DOI:** 10.18632/oncotarget.16918

**Published:** 2017-04-07

**Authors:** Xia Zhang, Qingyan Chi, Zhenhua Zhao

**Affiliations:** ^1^ Department of Respiratory Internal Medicine, The People's Hospital of Linyi City, Shandong, China; ^2^ Department of Neonatal, The People's Hospital of Linyi City, Shandong, China; ^3^ Department of Infectious Diseases, The People's Hospital of Linyi City, Shandong, China

**Keywords:** long non-coding RNA, *SPRY4-IT1*, lung adenocarcinoma, prognosis

## Abstract

Long non-coding RNAs (lncRNAs) play important role in a variety of biological processes, and are tightly associated with tumorigenesis and cancer prognosis. However, the potential power of lncRNA signatures in predicting survival of patients with lung adenocarcinoma (LUAD) has not been investigated. Here, we identified a four-lncRNA signature (*SPRY4-IT1*, *LINC00941*,*GPR158-AS1* and *KCNK15-AS1*) displaying prognostic values for LUAD using The Cancer Genome Atlas (TCGA) dataset. Based on the four-lncRNA signature, the LUAD patients can be classified into high-risk and low-risk groups with significantly different survival. We further validated the expression of *SPRY4-IT1* in LUAD tissues and corresponding normal lung tissues using quantitative real-time PCR in Chinese LUAD patients. The results showed that *SPRY4-IT1* was significantly up-regulated in LUAD tissues. Further analysis indicated that LUAD patients with high *SPRY4-IT1* expression have significantly poorer overall survival in Chinese LUAD patients. Moreover, knock-down of *SPRY4-IT1* can inhibit the lung cancer cell migration and invasion. The present work indicated that *SPRY4-IT1* may play a pivotal role in promoting tumor migration and invasion in LUAD. Our work implicates the promising effect of *SPRY4-IT1* on the prognosis of LUAD.

## INTRODUCTION

Lung cancer has become one of the most common malignancies, and the leading cause of cancer death worldwide [1]. More than 700,000 estimated new cases were diagnosed, and 60,000 patients died of lung cancer each year in China [2]. Lung adenocarcinoma (LUAD) is one of the predominant types of lung cancer in females and non-smoking males. Although therapeutic advances have significantly improved the prognosis of specific subgroups of LUAD patients, the overall survival rate of LUAD patients remains very low [3–5]. Therefore, novel cancer-specific marker is needed for LUAD patients, and it may enhance early tumor detection and treatment.

Long non-coding RNAs (lncRNA), a subclass of RNA transcripts longer than 200 nucleotides with little coding capacity, contribute to a significant regulatory information at transcriptional and epigenetic levels [6–9]. Accumulating evidence suggested that lncRNAs play important roles in the biological processes [10, 11], and dysregulation of lncRNAs is always associated with complex diseases, including cancers [12]. Many well-known lncRNAs have indicated tumor suppressive or oncogenic roles in various cancers, such as *H19* [13], *HOTAIR* [14], *MALAT1*, *MEG3* [15] and *PVT1* [16] etc. LncRNA expression functions as good indicator of tumor stage, implying the potential role as independent biomarkers for prognosis prediction in cancers.

Many recent works have been performed to detect lncRNA signatures to predict cancer survival [17–20], and we herein performed a comprehensive analysis for the lncRNAs expression and clinical outcome of LUAD patients based on TCGA dataset. A four-lncRNA signature displayed prognostic values for LUAD. Notably, *SPRY4-IT1* has been reported to be desregulated in several human cancers [11, 21, 22], such as hepatocellular carcinoma [23] and melanoma [24]. However, the underlying molecular mechanism of *SPRY4-IT1* remains unclear in LUAD. Here, we also examined the expression difference between LUAD tissues and normal lung tissues, and re-evaluated the clinical significance of *SPRY4-IT1* in Chinese LUAD patients. Moreover, we also evaluated the effects of *SPRY4-IT1* expression on tumor cell migration and invasiveness. This finding suggests the potential effects of *SPRY4-IT1* expression on LUAD prognosis.

## RESULTS

### Identification of prognostic lncRNAs associated with survival of LUAD patients

**Figure 1:**
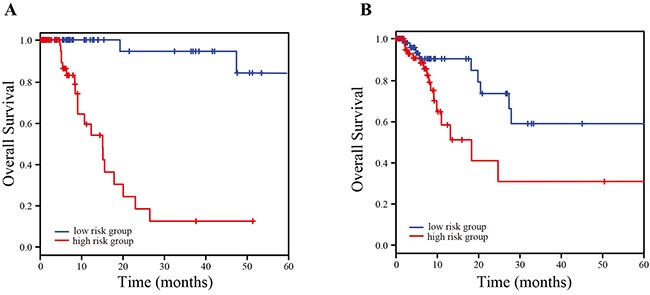
Kaplan-Meier estimation of overall survival of TCGA LUAD patients using the four-lncRNA signature **(A)** Kaplan-Meier curves for TCGA training set; **(B)** Kaplan-Meier curves for TCGA testing set.

We randomly divided 412 LUAD patients into a training dataset (n = 206) and a testing dataset (n = 206). At first, a univariate Cox regression analysis was carried out to evaluate the association between lncRNA expression and overall survival in the training dataset, and four lncRNAs were identified as prognostic lncRNAs (*P-value* < 0.001). Three lncRNAs (*SPRY4-IT1*, *GPR158-AS1* and *KCNK15-AS1*) with higher expression were associated with shorter survival, and the remaining lncRNAs (and *LINC00941*) with higher expression were associated with longer survival. Next, we constructed a risk-score formula according to lncRNA expression for survival prediction as follows: Risk score = (0.33 × expression of *GPR158-AS1*) + (0.12 × expression of *KCNK15-AS1*) + (0.27 × expression value of *SPRY4-IT1*) + (-0.22 × expression value of *LINC00941*). Based on this formula, we can calculate the risk score for each LUAD patient in the training dataset, and 206 LUAD patients in the training dataset were classified into a high-risk group (n = 103) and a low-risk group (n = 103) using the median risk score (-1.12) as the threshold. The result showed a significant difference in survival between the high-risk group and the low-risk group (*P-value* = 1.3E-09; Figure 1A). We next validated the survival prediction power of four-lncRNA signature, and 206 LUAD patients in the testing dataset were classified into a high-risk group (n = 126) and a low-risk group (n = 80) with the same threshold derived from the training dataset. The result showed that a significantly different survival between two groups (*P-value* = 2.6E-04; Figure 1B).

Next, we evaluated whether the survival prediction power of the four-lncRNA signature was independent of other clinical variables using entire TCGA dataset. We performed multivariable Cox regression analysis, and the result showed that four-lncRNA risk score was also significantly associated with overall survival after adjusted by age, tumor stage and other clinical variables (Table 1). This result demonstrated that the survival prediction of four-lncRNA signature was independent of other clinical variables for LUAD patients.

**Table 1:** Multivariable Cox proportional hazard model in TCGA LUAD dataset

Variables	Multivariable analysis		
	HR	95% CI	*P-value*
Age (Years)	0.87	0.82-1.02	0.24
Gender	0.73	0.47-1.49	0.36
Tumor size	0.92	0.86-1.01	0.32
Tumor stage	1.45	1.06-3.18	0.02
Risk score	1.68	1.24-2.66	0.001

### The expression of *SPRY4-IT1* in LUAD tissues and cell lines

**Figure 2:**
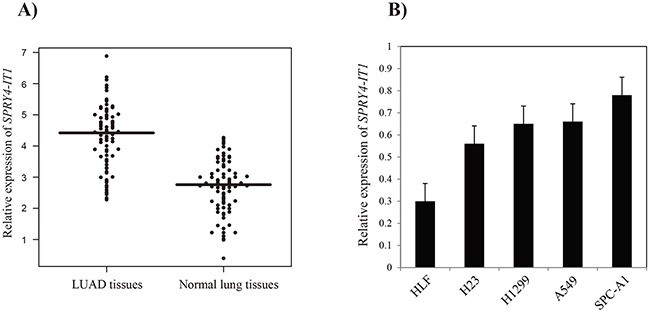
The relative expression levels of *SPRY4-IT1* in LUAD tissues and cell lines **(A)** The relative expression of *SPRY4-IT1* in LUAD tissues and adjacent normal lung tissues. The lines represent the means of the relative expression of SPRY4-IT1. **(B)** The relative expression of *SPRY4-IT1* in LUAD cell lines.

Among these four prognostic lncRNAs, *SPRY4-IT1* has been widely reported in many cancer types [21, 25], whereas none of the other three lncRNAs have been reported to be associated with human cancers. So, we attempt to further examine the expression pattern of *SPRY4-IT1* in LUAD patients. We measured the *SPRY4-IT1* expression level in the tumor tissues and adjacent normal lung tissues from 88 LUAD Chinese Han patients using quantitative RT-PCR. The result showed that *SPRY4-IT1* expression was significantly up-regulated in LUAD tissues (4.4±0.76) compared with that in the matched normal lung tissues (2.9±0.88, *P-value* < 0.0001, Figure 2A). Further analysis showed that *SPRY4-IT1* expressions in LUAD cell lines (H23, H1299, A549 and SPC-A1) were significantly higher compared with that in the normal lung fibroblast cell line HLF (Figure 2B).

### Clinical significance of *SPRY4-IT1* expression in LUAD

**Figure 3:**
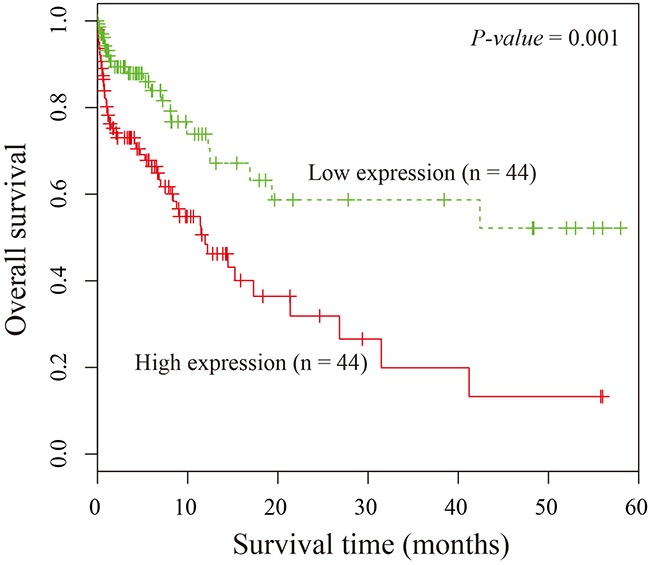
Kaplan-Meier survival curves of patients with LUAD based on *SPRY4-IT1* expression Patients in the higher expression group have significantly poorer prognosis than those in the lower expression group.

Next, we evaluated the relationship between *SPRY4-IT1* expression and clinicopathological characteristics in Chinese Han LUAD patients. All these patients were divided into two groups according to the median expression level of *SPRY4-IT1*, including a *SPRY4-IT1* high-expression group (n = 44) and a low-expression group (n = 44). Then, the correlation between *SPRY4-IT1* expression and pathological factors was examined. The result indicated that patients with higher*SPRY4-IT1* expression had larger tumor size (*P-value* = 0.012) and high histological grade (*P-value* < 0.001, Table 2). Kaplan-Meier survival analysis showed that the patients with higher *SPRY4-IT1* expression had a significantly poorer prognosis than those with low *SPRY4-IT1* expression level (*P-value* = 0.001, Figure 3). Univariate proportional hazard model showed that the histological grade and *SPRY4-IT1* expression level was prognostic predictor. Multivariate analysis further indicated that histological grade (*P-value* = 0.001) and *SPRY4-IT1* expression level (*P-value* = 0.001, Table 3) were independent prognostic factors for overall survival. This result suggests that *SPRY4-IT1* might be involved in the progression of LUAD and can be used to predict overall survival in Chinese Han LUAD patients.

**Table 2:** Clinicopathological associations of *SPRY4-IT1* expression in Chinese Han LUAD patients

*SPRY4-IT1* expression			
Variable	Low(n=44)	High(n=44)	*P-value*
Ages (years)			0.88
< 50	21	18	
≥ 50	23	26	
Gender			0.28
Male	19	24	
Female	25	20	
Tumor size			0.002
< 3 cm	20	13	
≥ 3 cm	24	31	
Smoking history			0.33
Non-smoker	18	15	
smoker	26	29	
Histological grade			<0.001
G1	11	7	
G2	15	10	
G3	18	27	

**Table 3:** Univariate and multivariate analyses of prognostic factors in LUAD

Variables	Univariate analysis			Multivariate analysis		
	Risk	95% CI	*P-value*	Risk	95% CI	*P-value*
Age (Years)	1.07	0.94-1.42	0.55	1.02	0.92-1.28	0.61
Gender	1.18	0.83-1.68	0.34	1.03	0.78-1.49	0.66
Tumor size	1.11	0.82-1.34	0.09	1.16	0.96-2.48	0.22
Histological grade	2.66	1.12-4.33	<0.001	2.05	1.11-4.08	0.001
*SPRY4-IT1*	2.21	1.16-3.88	<0.001	1.98	1.14-3.92	0.001

### Effect of *SPRY4-IT1* on LUAD cell migration and invasion

**Figure 4:**
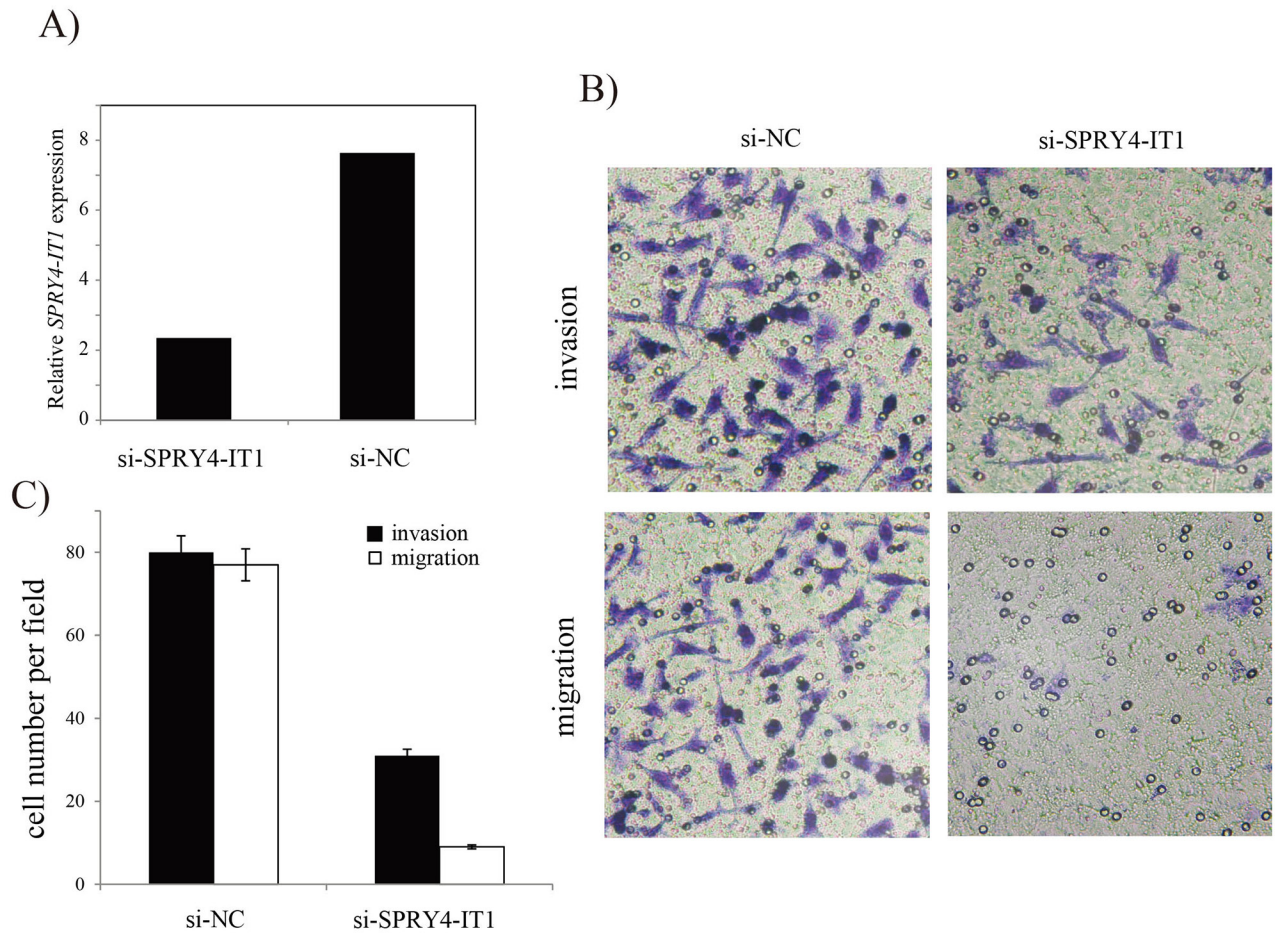
Effects of *SPRY4-IT1* on migration and invasion of A549 cell line **(A)** qRT-PCR analysis of *SPRY4-IT1* in A549 cells. **(B)** Invasion and migration assays of A549 cells and representative fields of migration and invasive cells. **(C)** Average number of invasive and migration cells per field from three independent experiments.

To investigate whether *SPRY4-IT1* regulates LUAD cell migration and invasion, *in vitro* functional analysis were performed. The result showed that knockdown of *SPRY4-IT1* in A549 cell by RNAi significantly inhibit cell migration and invasion (Figure 4), suggesting that *SPRY4-IT1* gene may function as an oncogene and promote the LUAD cell migration and invasion.

## DISCUSSION

LUAD is one of the most common tumors with a relative lower overall survival rate. Prognostic factor detection in LUAD patients is essential to predict patients’ survival and determine optimal therapeutic strategies. Recently, many efforts have been devoted to detect specific potential markers with prognosis in LUAD [26]. As a novel biomarker, the potential roles of long non-coding RNAs in predicting prognosis have been extensively identified. Until now, nearly more than 10000 human lncRNAs have been identified, which regulate most of the genes in human genome [9]. Due to the crucial role of lncRNAs regulation at the transcription level, lncRNAs are involved in many important biological functions, such as differentiation and proliferation. Furthermore, lncRNAs show aberrant expression patterns in various cancer types, and dysregulation of lncRNAs may influence the neoplastic progression and prognosis of human malignancies [27–29].

Using TCGA LUAD dataset, we detected a four-lncRNA signature in LUAD patients to predict overall survival. Among these four prognostic lncRNAs, *SPRY4-IT1* aroused our great interests. *SPRY4-IT1* is nested in the intron of the *SPRY4* gene. The expression level of *SPRY4-IT1* is highly correlated with *SPRY4* gene in many cancer types, suggesting that it plays an important regulatory role during the expression of *SPRY4* gene. Dysregulation of *SPRY4-IT1* has been reported to perform an important function in diverse cancer types, such as hepatocellular carcinoma and melanoma, etc. [22–24, 30]. The knockdown of SPRY4-IT1 expression results in defects in cell growth, decreased invasion, and increased rates of apoptosis in melanoma cells [30]. However, the underlying molecular mechanism of *SPRY4-IT1* in LUAD remains unclear. In this work, we investigated the expression level of *SPRY4-IT1* in LUAD and adjacent normal tissues. The results indicated that the expression of *SPRY4-IT1* was extensively up-regulated in LUAD tissues and cell lines. Further analysis showed that *SPRY4-IT1* could be used as a potential prognostic biomarker for LUAD. Functional characterization showed that knockdown of *SPRY4-IT1* could significantly inhibit cell migration and invasion, which indicates that SPRY4-IT1 might function as a oncogene in LUAD patients.

Taken together, our present work showed that *SPRY4-IT1* expression was up-regulated in LUAD and is tightly associated with cancer cell migration and invasion. Our data implicated for the first time that *SPRY4-IT1* expression was an independent prognostic factor of LUAD patients.

## MATERIALS AND METHODS

### TCGA LUAD datasets and lncRNA expression

The lncRNA expression dataset and corresponding clinical information of LUAD patients were derived from TCGA data portal (https://gdc-portal.nci.nih.gov/), and a total of 412 patients were involved in this work. The lncRNAs derived from TCGA were all annotated in GENCODE database [31] after removing the redundancy. All lncRNAs with their expression value lower than 0.3 were removed, and we finally obtained 14720 lncRNAs in 412 LUAD patients. The lncRNA expression level were defined as those with an average Reads Per Kilobase per Million mapped reads (RPKM) ≥ 0.1 across all 412 LUAD patients. Finally, we obtained expression profiles of 12730 lncRNAs in 412 LUAD patients.

### Statistical analysis

The 412 LUAD patients were randomly divided into a training set (n=206) and testing set (n=206). At first, the association between lncRNA expression and overall survival of LUAD patients was measured based on the training dataset using a univariate Cox regression analysis with a permutation test. Those lncRNAs with their P-values < 0.001 were considered to be prognostic lncRNAs. Then, a risk score model was constructed by including those prognostic lncRNAs, weighted by the estimated regression coefficients in the multivariable Cox regression analysis in the training set. Based on the risk score formula, LUAD patients can be classified into high-risk and low-risk groups for further Kaplan-Meier estimation.

### Patients and tissue samples

Surgical specimens of tumor tissues and paired normal adjacent tissues were obtained from LUAD patients who underwent surgery at the people's Hospital of Linyi City (Shandong Province, China) between April 2008 and Nov 2014. Informed written consents were obtained from all enrolled LUAD patients, and all LUAD patients had a complete five-year follow-up. All patients had never received any radiotherapy before surgery excision. All tissue samples were immediately frozen in liquid nitrogen after surgery.

### RNA extraction and RT-PCR

Total RNAs were extracted from tissues and cell lines using TRIzol reagent (Invitrogen, Carlsbad, USA) according to the manufacturer's protocol. The primer sequences of *SPRY4-IT1* and U6 were purchased from Applied Biosystems (ABI, Foster City, CA, USA). The sequence of primers were as following: *SPRY4-IT1* (forward: 5’-AGCCACATAAATTCAGCAGA-3’, reverse: 5’-GATGTAGTAGGATTCCTTTCA-3’). ABI 7500 was used to performed the qPCR work.

### Cell lines

Four human LUAD cell lines (H23, H1299, A549 and SPC-A1) and the human lung fibroblast cell line (HLF) were obtained from the American Type Culture Collection (Manassas, VA), and were cultured in RPMI 1640 medium (Gibco, USA) supplemented with 10% FBS.

### siRNA and plasmids DNA transfections

Small interfering RNA against *SPRY4-IT1* and nonspecific control siRNA were synthesized and transfected into cells using Lipfectamine 2000 transfection reagent. The sequence for the si-*SPRY4-IT1* was as following: si-SPRY4-IT1 (CCCAGAATGTT GACAGCTGCCTCTT).

### Cell migration and invasion assays

Cell migration and invasion ability was evaluated using specialized transwell chambers (8 µm pore; BD Biosciences). For invasion assay, a total of 2 × 10^5^ cells were added to the upper compartment of the chamber pre-coated with matrigel matrix. Medium containing 10% fetal bovine serum (FBS) was added to the lower chamber. After incubation for 24 hours, the non-invaded cells were removed from the upper surface with a cotton wool. The invasive cells were fixed with methanol, and then counted under a microscope. For migration assay, cells were added into upper chamber without pre-coated matrigel matrix. Images were captured by a microscope (5 independent fields per chamber), and the cells were counted blindly.
